# The American Medical Association

**Published:** 1882-07

**Authors:** 


THE AMERICAN MEDICAL ASSOCIATION.—The meeting at St. Paul seems to have been a very successful one, at least as far as numbers were concerned. It is said that the attendance was larger than at any previous meeting of the Association, except the one in this city in 1880. Much of the success of the meeting was due to the efficient Committee of Arrangements, with our energetic friend, Professor Stone, at its head. As one of our friends said on his return: "Stone is a regular *brick.*"

The following officers were elected for the ensuing year: *President*—Dr. John L. Atlee, of Pennsylvania. *Vice-Presidents*—Drs. Eugene Grissom, of North Carolina; A, J. Stone, of Minnesota; J. A. Octerlony, of Kentucky, and H. S. Orme, of California. *Treasurer*—Dr.

R. J. Dunglison, of Pennsylvania. *Librarian*—Dr. C. H. A. Kleinschmidt, of District of Columbia. *Chairman of Committee of Arrangements*—Dr. X. C. Scott, of Cleveland, O. *Assistant Secretary*—Dr. I. N. Himes, of Cleveland. *Judicial Council*—Drs. N. S. Davis, of Illinois; J. M. Brown, U. S. N.; X. C. Scott, Ohio; M. Sexton, Indiana; N. C. Husted, New York; Wm. Lee, Maryland; J. E. Reeves, West Virginia. *Committee on Necrology*—Dr. J. M. Toner, of Washington, chairman. *Committee on Publication*—Dr. W. B. Atkinson, of Philadelphia, chairman.

The chairmen of the various sections are as follows: Drs. J. H. Hollister, of Illinois, *Practice of Medicine;* W. F. Peck, of Iowa, *Surgery and Anatomy;* J. K. Bartlett, of Wisconsin, *Obstetrics;* Foster Pratt, of Michigan, *Medical Jurisprudence and State Medicine;* A. W. Calhoun, of Georgia, *Ophthalmology, Otology and Laryngology;* R. Blount, of Indiana, *Diseases of Children;* D. H. Goodwillie, of New York, *Dentistry.*

Dr. Truman W. Brophy, of Illinois, was appointed Secretary of the section of Dentistry.

The next meeting of the Association will be held at Cleveland on the first Tuesday in June, 1883.

